# Hepatitis C Virus Micro-Elimination Plan in Southern Italy: The “HCV ICEberg” Project

**DOI:** 10.3390/pathogens12020195

**Published:** 2023-01-28

**Authors:** Carmine Coppola, Loreta A. Kondili, Laura Staiano, Simona Cammarota, Anna Citarella, Mirko Pio Aloisio, Angelo Annunziata, Francesca Futura Bernardi, Aldo D’Avino, Michele D’Orazio, Marianna Fogliasecca, Mario Fusco, Federica Pisano, Adriano Vercellone, Elvira Bianco, Ugo Trama

**Affiliations:** 1 Department of Internal Medicine, Unit of Hepatology and Interventional Ultrasonography, OORR Area Stabiese, 80054 Gragnano, Italy; 2 Center for Global Health, Istituto Superiore di Sanità, 00161 Rome, Italy; 3 Department of Dentistry and Dental Prosthetic, UniCamillus-Saint Camillus International University of Health and Medical Sciences, 00131 Rome, Italy; 4 LinkHealth Health Economics, Outcomes & Epidemiology S.R.L., 80143 Naples, Italy; 5 Local Health Unit Naples 3 South, 80059 Castellammare di Stabia, Italy; 6 General Direction of Health Care & Regional Health System Coordination, Drug & Device Politics, Campania Region, 80143 Naples, Italy; 7 Cancer Registry, Local Health Unit Naples 3 South, 80100 Naples, Italy; 8 Pharmacy Unit, Local Health Unit Naples 3 South, 80059 Castellammare di Stabia, Italy; 9 Healthcare Direction, Local Health Unit Naples 3 South, 80059 Castellammare di Stabia, Italy

**Keywords:** administrative data, cascade of care, opportunistic screening, linkage to care, HCV micro-elimination

## Abstract

This study evaluates the feasibility of a local action program for HCV micro-elimination in highly endemic areas. Retrospective analysis: administrative and laboratory data (Local Health Unit, southern Italy) were integrated to quantize the anti-HCV-positive subjects not RNA tested and untreated HCV-infected subjects (2018–2022). Prospective analysis: all subjects admitted to a division of the LHU largest hospital (2021–2022) were tested for HCV, with linkage of active-infected patients to care. Overall, 49287 subjects were HCV-Ab tested: 1071 (2.2%) resulted positive without information for an HCV RNA test and 230 (0.5%) had an active infection not yet cured. Among 856 admitted subjects, 54 (6.3%) were HCV-Ab+ and 27 (3.0%) HCV RNA+. Of HCV-infected patients, 22.2% had advanced liver disease, highlighting the need for earlier diagnosis; 27.7% were unaware of HCV infection; and 20.4% were previously aware but never referred to a clinical center. Of these, 26% died and 74% received treatment. Our study emphasizes the value of an active HCV hospital case-finding program to enhance diagnosis in patients with several comorbidities and to easily link them to care. Our data strongly suggest extending this program to all hospital wards/access as a standard of care, particularly in highly endemic areas, to help HCV disease control and take steps in achieving the elimination goals.

## 1. Introduction

Infection with the hepatitis C virus (HCV) remains one of the leading causes of chronic liver disease and liver-related deaths worldwide. The availability of potent direct-acting antiviral therapies (DAAs) has provided an opportunity to reverse the rising burden of HCV-related complications [1,2]. At the beginning of 2020, 56.8 million viraemic HCV infections were estimated globally [3]. Although this number represents a decline from 2015, the estimates suggest that we are not currently on track to achieve global elimination targets by 2030 [4]. Furthermore, the COVID-19 pandemic has resulted in many hepatitis elimination programs slowing or stopping altogether [5].

Italy had the highest number of HCV liver-related deaths and the highest number of treated patients with DAAs in Europe. [6,7]. After an initial phase characterized by the prioritization of the patients with more severe liver disease (i.e., cirrhosis), in 2017, universal access to DAA treatment for those diagnosed with hepatitis C was introduced, covered by the National Health System (NHS) independently of severity of liver damage and without any sociodemographic or economic restriction [8]. Therefore, Italy was listed as a country on track to achieve the WHO goals in 2018 [9]. In subsequent years, the decrease in treatment rate in 2019 and almost an interruption of antiviral treatment during the COVID-19 pandemic have resulted in Italy falling behind in achieving WHO targets. Screening strategies are, therefore, necessary to sustain treatment rates required for Italy to achieve HCV elimination. The best cost-effectiveness strategy in Italy was defined as testing the general population birth cohort 1948–1988, starting first with the younger populations and the key populations (i.e., intravenous drug users and people detained in prison) at any age [10]. At the beginning of 2021, a new nationwide action has been implemented consisting of a dedicated fund for screening of active drug users and detainees without age restriction and birth cohort 1968–1988 [11]. This is only the first step towards elimination, and healthcare authorities need to target the whole birth cohort 1948–1988, estimated to maintain the high HCV infection burden yet not cured in Italy. A key aspect of the HCV elimination strategy is to identify interventions according to local needs.

Engagement in the continuum of HCV cascade care includes several steps: patients tested for HCV antibodies (HCV-Ab); patients tested for HCV-Ab test and confirmatory RNA; HCV RNA-confirmed patients who are in medical care; HCV RNA-confirmed patients who are in care receive antiviral treatment; monitoring patients during and after treatment [10,11]. Loss to follow-up occurs in each step of the care cascade and is a barrier to HCV elimination. The extent of this issue remains unclear especially in the late DAA era.

The purpose of this paper is to describe the results of a local micro-elimination plan, namely the “HCV ICEberg” project, developed in a local health unit (LHU) of the Campania region (South of Italy), an area previously known to have had a high prevalence of HCV infection. The HCV ICEberg project aimed to estimate the burden of anti-HCV-positive tests that did not complete the entire diagnostic and potential linkage-to-care pathway, with the final goal to evaluate the value of a hospital screening program in capturing HCV active infections, ensuring linkage to care and viral eradication.

## 2. Methods

The HCV ICEberg project was carried out in the LHU Napoli 3 Sud of the Campania region. The LHU is responsible for the management of healthcare services that include prevention departments and hospital centers for a residential population of 1,070,000 inhabitants, 13 districts and eight hospital facilities. The project was structured in two phases: a retrospective and a prospective phase.

Retrospective analysis was realized by integrating the administrative and laboratory data flow of the LHU and aimed to estimate both the number of patients with positive anti-HCV test not tested for RNA and the number of untreated HCV-infected patients.

The prospective evaluation was based in analyzing all individuals admitted to the General Medicine division of the “San Leonardo Hospital” (the largest hospital of the LHU), aiming to capture HCV-positive cases and evaluate their care cascade up to viral eradication.

### 2.1. Retrospective Phase

All data were linked through a unique individual identification code properly anonymized to respect the subject’s privacy. To guarantee the patients’ privacy, an anonymous univocal numeric code was assigned to each study subject, in full compliance with the European General Data Protection Regulation (GDPR) (2016/679). No identifiers related to patients were provided to the authors. The civil registry was linked with the pharmaceutical database, providing data on all the prescriptions reimbursed by the National Health System as ATC (Anatomical Therapeutic Chemical) code and prescription date, and with the Laboratory Information System (LIS) of all eight hospitals in the LHU, including code and date of diagnostic test.

We extracted all anonymized individuals 18 years and older and alive on 30 June 2022, with an HCV-Ab test result from the LIS between 1 January 2018 and 30 June 2022. A positive HCV-Ab test result during the study period was identified. The presence of a positive HCV RNA test classified an individual as HCV. Quantitative HCV RNA tests were considered positive if the viral load was 15 IU/mL or higher. For our purposes, we identified the subjects who had a positive HCV-Ab test but no information for an HCV RNA test during the study period and those who had a positive HCV RNA test as the last result available in the LIS. The subjects already treated with DAA from January 2015 to June 2022 were excluded. Finally, the following study cohorts were identified: i) individuals with positive HCV-Ab but no information for an HCV RNA test during the study period (HCV-Ab+ subjects not tested for RNA); ii) individuals with a positive HCV RNA test but not yet treated with DAA (untreated HCV RNA+ subjects).

### 2.2. Prospective Phase

All consecutive patients admitted for any cause to the General Medicine division of the hospital between September 2021 and October 2022 were tested for HCV-Ab as routine examination. All anti-HCV-positive individuals were asked if they were aware of the HCV infection and if they were already treated. For each anti-HCV-positive patient, also previously treated with DAA, an HCV RNA test was performed during hospitalization. If positive, the program continued as follows: if the patients were still hospitalized, they completed full liver function tests and DAA treatments were immediately started; if the patients were discharged from hospital, they were recalled by a specialist of the general medicine unit to initiate DAA treatment as soon as possible after the hepatological evaluation.

### 2.3. Ethics

This project was submitted to and approved by the local ethics committee of the LHU. This study was conducted in accordance with the guidelines of the Declaration of Helsinki and the principles of good clinical practice. The patients’ data were collected and conserved according to the General Data Protection Regulation (EU) 2016/679, as required for retrospective evaluations of clinical practice. Informed consent was obtained from all subjects involved in the prospective phase of the study.

### 2.4. Statistical Analysis

Descriptive statistics were used to report the distribution of patients according to the birth groups (before 1947, 1948–1968, 1969–1989, after 1990) and gender. All variables were expressed as absolute numbers and percentages.

## 3. Results

### 3.1. Retrospective Analysis

**Figure 1:**
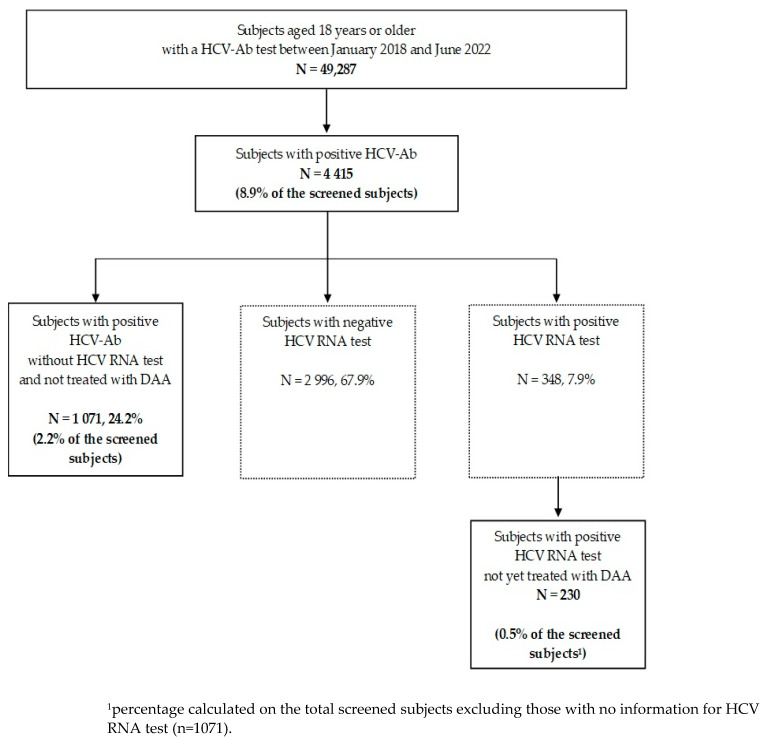
Flow chart of the retrospective study.

Between January 2018 and June 2022, 49,287 subjects performed an HCV-Ab test: 4415 (8.9% of the overall screened subjects) had a positive HCV-Ab test (Figure 1).

Among these, 1071 individuals (24.2% of all HCV-Ab+ subjects) had no information for an HCV RNA test during the study period, equal to 2.2% of the total screened subjects. Of those, 53.7% were female, 63.0% were born before 1947, and 27.4% between 1948–1968 (Table 1).

Among those tested for HCV RNA, 348 (7.9% of all HCV-Ab+ subjects) had a positive HCV RNA result as the last value available in the LIS. Finally, a cohort of 230 individuals with active infections not yet treated with DAA were identified. The prevalence of active infections was 0.5% of the overall screened population (excluding 1071 HCV-Ab+ subjects not tested for HCV RNA) (Figure 1). Of these, 53.5% were female, 45.6% were born before 1947, followed by 42.2% born between 1948–1968 (Table 1).

### 3.2. Prospective Analysis

**Figure 2:**
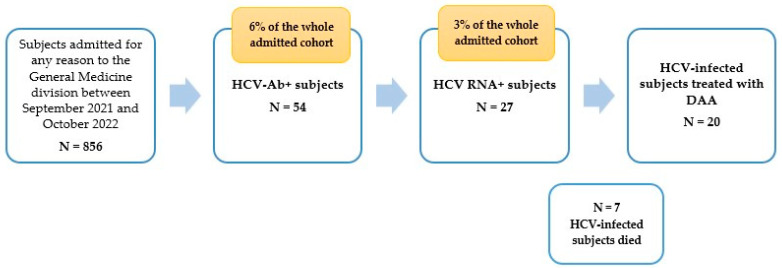
Flow chart of the hospital case-finding program (prospective phase).

Of 856 subjects admitted for any reason to the General Medicine division of the hospital, between September 2021 and October 2022, 54 patients (6.3%) tested anti-HCV positive (Figure 2).

Some 51.9% were males and 64.8% were born before 1947, followed by 27.8% born between 1948–1968 (Table 2).

Of 54 anti-HCV-positive individuals, 27.7% (15/54) were unaware of their serological status, 20.4% (11/54) were previously aware of HCV infection but never referred to a clinical center, and 24.1% (13/54) were already treated with DAA.

We found 27 subjects with an active infection, equal to 3% of the whole cohort admitted to the hospital division (Figure 2). Some 59.3% of viraemic patients were female and 70.4% were born before 1947, 22.2% between 1948–1968, 3.7% between 1969–1989, and 3.7 after 1990. A total of 12 HCV-infected patients (44.4%) had hypertension, eight (29.6%) cardio-cerebrovascular disease, six (22.2%) diabetes, six (22.2%) advanced liver disease (five decompensated cirrhosis and one hepatocellular carcinoma), and one (3.7%) had HIV co-infection (Table 1). A total of 26% (7/27) of viraemic patients died and 74% (20/27) were treated with DAA.

## 4. Discussion

In the present study, we reported the results of a local micro-elimination program based on a multi-intervention approach. This model allows researchers to perform health services research in patients with HCV using routinely collected administrative healthcare data and to capture positive cases—unaware of their status or not yet linked to care— through a hospital case-finding program.

Our retrospective analysis of administrative healthcare data shows that 2.2% of the screened population did not complete the entire diagnostic pathway (24.2% of all anti-HCV-positive individuals) and 0.5% had an active infection not yet cured with DAA. As reported in several studies, loss of patients may occur in all steps of the HCV care cascade starting from HCV testing to referral and treatment [12]. Loss to follow-up prevents patients from receiving the care they need to be cured of their infection. Results from recent experience in the Campania region, which involved 44 general practitioners, found that even after identification, 42% of patients were not referred to a specialist center for further evaluation, and only 39% were treated. The authors reported similar findings both in patients with an already known HCV diagnosis and those with a newly diagnosed HCV infection, suggesting the need for greater outreach, awareness, and education among general practitioners (GPs) in order to enhance HCV testing and linkage to care [13]. The “Telepass project”, based on a hospital recall strategy of anti-HCV patients, revealed that 18.69% of recalled patients, even if aware of their serological status, did not perceive the possibility of being HCV infected as a harmful condition that ought to be further investigated [14].

In the retrospective analysis, a large proportion of anti-HCV-positive individuals had negative serum HCV RNA. Southern Italy has been in the past highly endemic for HCV infection, and this finding clearly indicates the role of antiviral treatment (DAA or interferon-based treatment) or, even if in small proportion, spontaneous viral clearance during the natural course of HCV infection [15,16].

Through the hospital case-finding program, we captured 54 anti-HCV-positive patients who were admitted to the general medicine division for any reason between September 2021 and October 2022. About 28% of anti-HCV patients were unaware of their serological status and 20% were previously aware of HCV infection but never referred to a clinical center. After HCV RNA testing, we found 27 patients with active HCV infection: 26% died and 74% were treated with DAA. These findings add to recent evidence highlighting that an active HCV case-finding strategy in the hospital setting is an important opportunity to enhance HCV diagnosis and to reduce barriers to HCV cascade care and cure. Rosato et al. showed the opportunistic hospital screening in admitted patients with various comorbidities as a feasible strategy to diagnose and subsequently easily link to care the infected patients [17]. Messina et al. reported the efficacy of an innovative model of HCV micro-elimination in a hospital setting through a retrospective analysis of internal databases and recalling patients with positive HCV-Ab [18]. An important finding of our study was that the total of viraemic patients who did not die were treated with DAA. This positive result was probably due to the fact that the patients’ approach was managed by physicians with proven experience in counseling and HCV management, and therefore were able to provide all the useful information to persuade anti-HCV-positive patients of the importance of infectious disease evaluation.

In regard to the birth cohorts, in line with previous research, we found that more than 70% of viraemic patients were born before 1947 and around 22% between 1948–1968 [17]. This implies that diagnosis and linkage to care in birth cohorts of subjects with several comorbidities—and frequently with an advanced liver disease—are strictly required, in addition to the mass screening of the 1969–1989 birth cohort of individuals unaware of their infection status, already covered by a dedicated funding to achieve HCV elimination in Italy. In these last individuals, opportunistic HCV screening in hospital is only one of several ways to reach them. Other settings are also activated for active HCV screening in different Italian regions because the young population, as confirmed by our data, have less access to the hospital compared to the older population with several comorbidities. Currently, testing for hepatitis C can be performed for several reasons, such as a survey for abnormal liver function, preoperative assessment, blood donation, or routine evaluation in the hospital. Despite anti-HCV-positive patients clearly needing HCV confirmatory testing, a high proportion of anti-HCV-positive patients did not follow further investigation in the hospital routine. It is essential to raise awareness even among healthcare professionals about infection, appropriate hepatitis testing, referral, and care. In addition, reflex testing of active infections of individuals unaware of chronic HCV status is indicated with specific regard in hospital settings, in that it reduces the timing required to diagnosis and potential linkage to care. It is strongly requested and is shown a cost-effective intervention [19] to avoid the loss of patients in the HCV care cascade.

Interestingly, in our experience, 22% of viraemic HCV patients had advanced liver disease (i.e., decompensated cirrhosis or hepatocellular carcinoma), highlighting the need for earlier diagnosis. Our findings confirm other research that underscores late diagnosis and presentation to specialist viral hepatitis care as an important issue within the healthcare system [20]. Additionally, HCV-related disease inflicts a huge economic and clinical burden, even as a result of HCV-related extrahepatic comorbidities [21]. Early eradication of HCV, before it progresses to advanced liver disease, could reduce these burdens [22].

Chronic HCV infection is a multisystemic disease, and data from our study and other data reported by hospital settings in different geographical areas in Italy suggest that, in order to achieve the overall HCV liver and extrahepatic disease burden, presence of HCV chronic infection should be checked as a standard of care in admitted patients who are frequently affected by different comorbidities that could have been caused or impacted by HCV chronic infection. Eradication of HCV infection has shown significant improvement of hepatic and extrahepatic manifestation [23].

Our study has some potential limitations. Our retrospective analysis takes into account only the HCV testing performed by the hospital public health laboratories of the LHU. In particular, the proportion of the screened population that did not complete the entire diagnostic pathway could be overestimated because the patients could have had an HCV RNA test performed outside the LHU. However, the loss of patients in the diagnostic pathway has been demonstrated by several realities [12]. Moreover, the untreated HCV RNA individuals may have received the DAA treatment after June 2022; however, not immediately after being diagnosed. Special populations (such as people who inject drugs) notably at high risk for HCV infection are unlikely to be represented in this study because they are typically young and less likely to turn up at healthcare facilities. However, these populations are addressed by the screening law decree, where the hospital screening is only one of the ways to approach them. Finally, the generalizability of our findings should be considered with caution since the hospital case-finding program enrolled patients over a 1-year period in a single division, which could have influenced the high prevalence of active infection. Although data from other experiences in Southern Italy conducted in more than one unit have shown similar results, larger studies in other Italian regions should be carried out to evaluate the feasibility and to confirm the results of opportunistic screening as a useful tool for identification of individuals with active infection not diagnosed or diagnosed but not yet treated. Despite the fact that the prevalence of infection could be dissimilar in different geographical areas in Italy and thus not generalizable, hospital screening is suggested by the screening law decree of the Ministry of Health and has been implemented in several Italian regions. Knowledge-sharing across regions, also using data from this study, can improve screening practices and encourage improved and innovative methods for convincing these individuals to get tested.

## 5. Conclusions

In conclusion, the study demonstrates the feasibility of a local action program for eliminating HCV. Patient loss is a problem in each step in the HCV care cascade, even in the current era where highly effective and tolerable treatments are available. Moreover, our study emphasizes the value of an active HCV case-finding program in hospital settings to enhance the diagnosis in patients with various comorbidities and to subsequently easily link them to care, suggesting HCV testing as a standard of care, particularly in highly endemic areas. Our data strongly suggest extending this program to all hospital wards/access as a standard of care, particularly in highly endemic areas, in order to help HCV disease control and steps in achieving the elimination goals, together with other micro-elimination programs involving special populations.

## Figures and Tables

**Table 1:** Baseline characteristics of the study cohorts using administrative data (retrospective phase).

Characteristics	HCV-Ab+ Subjects Not Tested for RNA N = 1 071		Untreated HCV RNA+ Subjects N = 230	
	N	%	N	%
Sex				
Male	496	46.3	107	46.5
Female	575	53.7	123	53.5
Year of birth				
Before 1947	675	63.0	105	45.6
1948–1968	293	27.4	97	42.2
1969–1989	81	7.6	25	10.9
After 1990	22	2.0	3	1.3

**Table 2:** Demographic and clinical characteristics of the HCV-Ab-positive subjects and HCV RNA-positive subjects (prospective phase).

Characteristics	HCV-Ab+ Subjects N = 54		HCV RNA+ Subjects N = 27	
	N	%	N	%
Sex				
Male	28	51.9	11	40.7
Female	26	48.1	16	59.3
Year of birth				
before 1947	35	64.8	19	70.4
1948–1968	15	27.8	6	22.2
1969–1989	3	5.6	1	3.7
After 1990	1	1.9	1	3.7
Comorbidity				
Hypertension	20	37.0	12	44.4
Diabetes	18	33.3	6	22.2
Cardio-cerebrovascular disease	16	29.6	8	29.6
Decompensated cirrhosis	15	27.8	5	18.5
Chronic obstructive pulmonary disease	13	24.1	7	25.9
Chronic renal failure	12	22.2	5	18.5
Anemia	8	14.8	3	11.1
Hepatocellular carcinoma	5	9.3	1	3.7
Other cancer	4	7.4	3	11.1
Dyslipidemia	5	9.2	2	7.4
Human Immunodeficiency Virus	1	1.9	1	3.7
Dementia	1	1.9	1	3.7

## Data Availability

Restrictions apply to the availability of these data. The readers may contact the authors to access these data.
